# Fasting-Induced Molting Impacts the Intestinal Health by Altering the Gut Microbiota

**DOI:** 10.3390/ani14111640

**Published:** 2024-05-31

**Authors:** Hao Zhang, Yihui Zhang, Yujie Gong, Jun Zhang, Donghua Li, Yadong Tian, Ruili Han, Yujie Guo, Guirong Sun, Wenting Li, Yanhua Zhang, Xinlong Zhao, Xiaoran Zhang, Pengyu Wang, Xiangtao Kang, Ruirui Jiang

**Affiliations:** 1 College of Animal Science and Technology, Henan Agricultural University, Zhengzhou 450002, China; zh15890617160@163.com (H.Z.); a7097179742024@163.com (Y.Z.);; 2 Key Laboratory of Livestock and Poultry Resources (Poultry) Evaluation and Utilization, Ministry of Agriculture and Rural Affairs, Zhengzhou 450002, China

**Keywords:** fasting-induced molting, intestinal health, microbiota, metabolome

## Abstract

**Simple Summary:**

In poultry production, fasting can remodel ovarian function and thereby delay the aging process of chickens. However, this approach may induce various stresses on the intestinal health of chickens. So, intestinal injury, the microbiome, and the metabolome were analyzed individually and integrated to elucidate the impact of the intestinal flora on intestinal injury during fasting-induced molting (FIM). The findings revealed that fasting led to intestinal villus atrophy, inflammation in the intestines, and disruption of gut microbiota and metabolites. During the fasting period, intestinal injury is associated with an increased abundance of *Escherichia_Shigella* and related metabolites Citrulline and Sterobilin, as well as a decreased abundance of *Ruminococcaceae_UCG-013* and *Lactobacillus* and related metabolites Lanthionine and reduced Glutathione. This provides an experimental basis for improving the gut microbiota and reducing intestinal inflammation during the FIM process.

**Abstract:**

Fasting-induced molting (FIM) is a common method used to improve the laying performance of aged laying hens. Nevertheless, this approach may impose various stresses on chickens, such as disruptions in intestinal flora and inflammation issues within the intestines. However, the impact of an imbalance in intestinal flora on intestinal health during the FIM process remains elusive. Therefore, intestinal injury, the microbiome, and the metabolome were analyzed individually and integrated to elucidate the impact of the intestinal flora on intestinal health during the FIM process. The findings indicated that fasting resulted in a notable reduction in villus height and villus/crypt ratio, coupled with elevated levels of intestinal inflammation and permeability. During the fasting period, microbiota compositions changed. The abundance of *Escherichia_Shigella* increased, while the abundance of *Ruminococcaceae_UCG-013* and *Lactobacillus* decreased. *Escherichia_Shigella* was positively correlated with Citrinin and Sterobilin, which lead to intestinal inflammation. *Ruminococcaceae_UCG-013* and *Lactobacillus* exhibited positive correlations with Lanthionine and reduced Glutathione, thereby reducing intestinal inflammation. This study screened the intestinal probiotics, *Ruminococcaceae UCG-013* and *Lactobacillus*, that influence gut health during the fasting period, providing an experimental basis for improving gut microbiota and reducing intestinal inflammation during the FIM process.

## 1. Introduction

In nature, birds need to undergo a natural molting process before winter arrives, shedding old feathers and growing new ones to cope with the cold winter climate. Molting usually takes place in the autumn, during which birds greatly reduce their food intake, experience a decrease in ovarian function, and their weight drops by around 30–40% [1]. The natural molting period lasts approximately 3–4 months and is associated with reduced egg production rates and decreased eggshell quality in hens [2]. For commercial egg-laying hens, their economic value will significantly decrease after undergoing natural molting. In order to maintain an uninterrupted supply of eggs, many farmers opt to sell and replace hens before they naturally molt. Induced molting is an alternative method capable of prompting hens to enter a molting state by modifying their environment, nutrition, and hormones. This process promotes the degeneration and remodeling of the reproductive system, which allows them to begin a new laying cycle more quickly [3]. Induced molting has been demonstrated to address difficulties in introducing and maintaining laying hens, prolonging the breeding and laying hens’ lifespans, and improving subsequent egg production and eggshell quality [3,4]. Furthermore, this practice has the potential to impact the immune function of hens, reduce breeding costs, and meet the demands of the egg market [4,5]. Effectively implementing induced molting is an important measure for the egg industry to deepen the promotion of grain conservation actions, advance full-chain conservation and reduction, and alleviate the food security issues caused by competition for food between humans and animals.

Now, the four main methods commonly utilized for inducing molting are farming, hormone administration, chemical intervention, and a combination of these methods. The farming method, known as the fasting method, capitalizes on the reduced feed intake characteristic during the natural molting phase of poultry. It involves a period of fasting, water restriction, and adjustment of the lighting schedule to stimulate the flock, facilitating a prompt transition of laying hens into a new egg-laying cycle [3,6]. The hormone method entails injecting adrenal hormones, thyroid hormones, and progesterone into the muscle of poultry, resulting in a hormonal imbalance, perturbing the physiological equilibrium, and prompting laying hens to cease egg production and undergo molting [7,8]. However, the hormone-induced molting results in poorer production performance in the laying hens in the second egg-laying cycle, leading to its limited practical application. The chemical method entails adjusting the amount of chemical agents in the feed to disrupt the metabolic balance in the body, thereby inducing molting in laying hens [5]. The combined method, which integrates fasting and chemical techniques, can expedite molting in poultry and lower mortality rates, but it does not result in complete molting. In contrast to alternative molting methods, fasting-induced molting (FIM) is characterized by shorter durations, reduced mortality rates, extended egg-laying peaks, and decreased farming expenses, rendering it the prevalent molting approach in egg production [5].

In nature, birds molt and incubate at the same time. Birds exhibit a spontaneous decrease in food intake during the incubation period. Throughout the incubation period, hens experience a decrease in weight of approximately 20%, a decline in sex hormone levels, and the degeneration of reproductive organs [9,10]. Following the hatching of the poultry, the hen progressively resumes feeding, undergoes physiological rearrangement, molts, and initiates a new egg-laying cycle. Consequently, voluntary fasting linked to incubation represents a physiological adaptation of birds in environments where simultaneously caring for offspring and foraging are incompatible [5]. FIM can significantly enhance the production performance and egg quality of hens in the late laying period, thereby prolonging their laying cycle [3,6]. FIM can revitalize the reproductive system of mature hens, restore ovarian function, and subsequently improve egg-laying performance [3]. However, fasting may also result in substantial physiological stress on the gastrointestinal tract, leading to various issues concerning intestinal health.

Fasting as a primary treatment modality lacks sufficient energy sources and may result in the development of intestinal microecological disorders and metabolic disturbances, compromised intestinal barrier function, and decreased nutrient absorption efficiency [11]. The interaction of intestinal microbiota and their metabolites with the immune system plays a crucial role in preserving both intestinal health and immune homeostasis. In White Leghorn hens and Ross 308, fasting may result in a progressive decrease in the height of intestinal villi, accompanied by the development of large lysosomal autophagic vacuoles in the intestinal epithelial cells [12,13]. The injury to the intestinal villi affects the maturation and activation of dendritic cells and T cells, as well as influences intestinal barrier function, subsequently triggering immune responses in the intestine [14,15]. Intestinal epithelial cells and immune cells collaborate in defending against intestinal pathogens. Injury to intestinal villi may impair immune cells’ capability to eliminate pathogens. During the fasting period, the susceptibility of laying hens to Salmonella significantly increased, and the expression of the virulence regulatory gene hilA in Enteritis Salmonella was upregulated [16]. The concentrations of acetic acid, propionic acid, and total volatile fatty acids in the cecum of laying hens during the fasting period were significantly lower than those in normally fed hens during the FIM process [17]. Acetic acid, propionic acid, and volatile fatty acids have the ability to inhibit Salmonella infection and lessen intestinal damage [14]. Currently, the relationship between changes in the intestinal microbiota and their metabolites during the FIM process and intestinal health is not clear.

To fully comprehend and utilize the effect of gut microbiota and their metabolites on gut health during the FIM process in laying hens, intestinal injury, microbiome, and metabolome were analyzed individually and integrated. This study establishes a foundation for investigating how the gut microbiota impacts intestinal inflammation during the FIM process, aiming to offer a theoretical framework for targeted enhancement of intestinal health in subsequent research.

## 2. Materials and Methods

### 2.1. Ethics Statement

All studies involving Houdan hens were approved by the regulators for the administration of affairs concerning experimental animals (Revised Edition, 2017). The experiments conducted in this study were approved by the Institutional Animal Care and Use Committee of Henan Agricultural University (Permit Number: 11-0099).

### 2.2. Animals and Experimental Design

**Figure 1:**
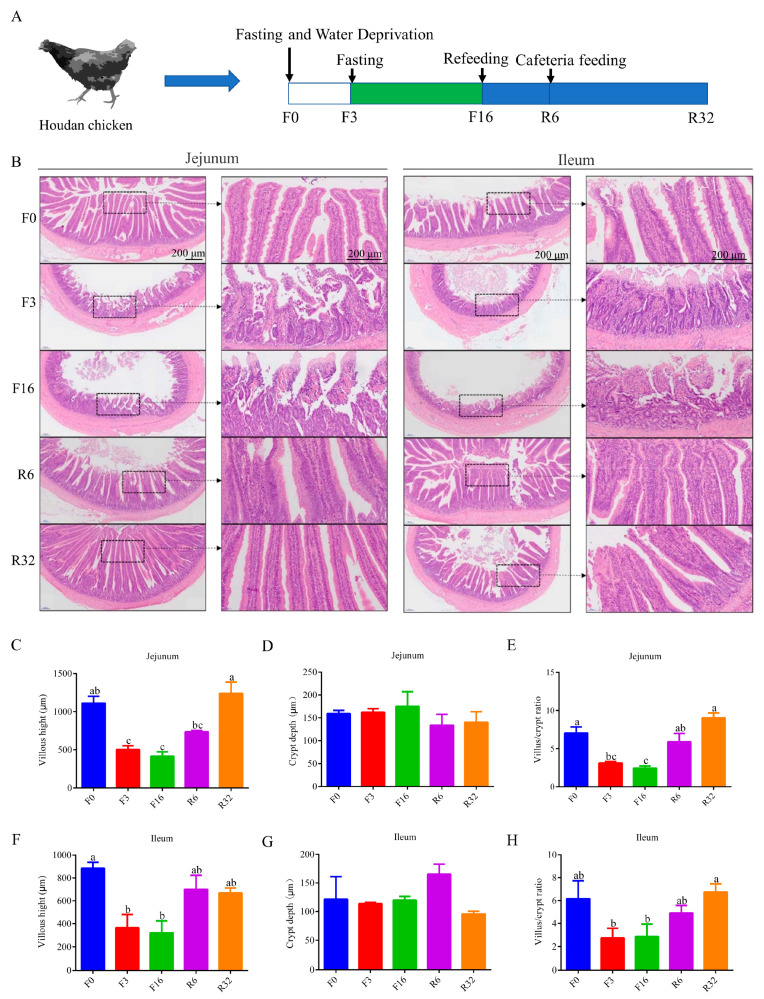
Morphological change in intestine in laying hens during the FIM process. (**A**) Experimental scheme of sampling during the FIM process. (**B**) H&E staining of the jejunum and ileum. Scale bars represent 200 μm. The right images of the jejunum and ileum were the enlarged parts of the left images. (**C**) Villus height of the jejunum. (**D**) Crypt depth of the jejunum. (**E**) Villus/crypt ratio of the jejunum. (**F**) Villus height of the ileum. (**G**) Crypt depth of the ileum. (**H**) Villus/crypt ratio of the ileum. The data were shown as the mean ± SEM. Data with different superscript letters (a, b, c) were significantly different (*p* < 0.05).

The experimental animals were provided by the Poultry Germplasm Resource Factory of Henan Agricultural University, Yuanyang County, Henan Province. The study was conducted from September 2021 to January 2022, maintaining the temperature in the chicken house at 18–25 °C and the humidity at 60%–70%. Ninety Houdan chickens with similar body weights, 72 weeks of age, and a laying rate of 60% were selected. They were evenly divided into 9 replicates (10 hens/replicate). The feed used for feeding is pre-laying feed-525 produced by Henan Tiankang Hongzhan Industrial Co., Ltd (Zhengzhou, China). The chosen hens were moved to the experimental poultry facility and given a 7-day acclimation period to adjust to the new environment before starting the fasting-induced molting process. Molting was induced by a 16-day fast, including an initial 3 days without water until egg production ceased entirely. Concurrently, the chickens were subjected to 8 h of light followed by 16 h of darkness during the fasting process. Fasting was discontinued when the body weight decreased by 30%, and the experiment concluded upon weight restoration to the pre-fasting level. The changes in body weight and egg production rate of laying hens during this fasting-induced molting process have been described in previous articles by our research team [3]. The FIM experiment focused on five key time points (Figure 1A): the day before fasting (F0), the 3rd day (F3), and the 16th day (F16) of fasting, the 6th day (R6), and the 32nd day (R32) of refeeding [18]. At each time point, six chickens are sampled, and their cecal contents are randomly paired and mixed to create three groups for 16S sequencing. Concurrently, the cecal contents of these 6 chickens are individually sampled for metabolome sequencing.

### 2.3. Determination of Intestinal Morphology

Jejunum and ileum tissue samples were embedded in paraffin and then cut into 4 μm thick sections with a rotary slicer. The sections were stained with hematoxylin and eosin (H&E) [19]. A total of 10 villi with a complete shape and straight direction in each tissue section were selected, and the villus height and crypt depth were measured using CaseViewer. The mean value was used as the measurement value of the sample, and the villus/crypt ratio was calculated.

### 2.4. Real-Time Quantitative PCR Analyses

Total RNA from jejunum and ileum tissue was extracted using TRIzol reagent (Invitrogen, Carlsbad, CA, USA). RNA concentration was measured using a NanoDrop ND2000 spectrophotometer (NanoDrop Products, Wilmington, NC, USA), according to the manufacturer’s instructions. High-quality RNA samples (concentration > 500 ng/µL, OD: 260/280 = 1.8–2.2, OD: 260/230 = 1.8–2.2) were used to reverse transcribed. RNA was reverse transcribed into cDNA using a Prime Script RT Reagent Kit (TaKaRa, Dalian, China). The resulting product was diluted to a concentration of 500 ng/mL and stored at −20 °C until needed. Real-time quantitative PCR (qRT-PCR) was performed to analyze the mRNA levels of *IL1β*, *IL8*, *NF-kB1*, *Occludin-1*, *ZO-1*, and *Claudin-1* using the 2^−ΔΔCT^ method. qRT-PCR was conducted using an SYBR Premix Ex Taq II kit (Takara, Dalian, China) on a LightCycler 96 Real-Time PCR system (Roche, Basel, Switzerland). The reaction components included 1 μL cDNA product, 5 μL 2× SYBR Premix Ex Taq II, 0.5 μL specific primer (10 mmol/L), and 3 μL deionized water. The qRT-PCR cycling conditions consisted of a total of 40 cycles: 95 °C for 5 min followed by 40 cycles at 95 °C for 30 s, 60 °C for 30 s, 72 °C for 30 s, and a final cycle at 72 °C for 5 min. The primer sequences for these genes can be found in Table S1.

### 2.5. DNA Extraction and 16S rDNA Sequencing Analysis

After extracting genomic DNA from cecal samples, the V3-V4 region of the 16S rDNA was amplified using specific primers with barcodes. The PCR sequences were performed using the primers 341F (CCTACGGGNGGCWGCAG) and 806R (GGACTACHVGGGTATCTAAT). The purified amplicons, which are the amplified products, were then ligated with sequencing adapters to construct sequencing libraries. Subsequently, Illumina sequencing was performed. After obtaining raw reads from sequencing, we filtered out low-quality reads and performed assembly and filtering steps to obtain high-quality reads. These reads were then clustered to generate operational taxonomic units (OTUs). Based on the analysis workflow, we conducted various analyses, including alpha/beta diversity, taxonomic composition, indicator species analysis, and PICRUSt2 analysis.

### 2.6. Cecal Contents Metabolite Analysis

In this study, cecal contents were thawed on ice and extracted using an 80% methanol buffer. The extracted samples were subsequently analyzed using liquid chromatography–mass spectrometry (LC-MS), according to the system’s instructions [20,21]. The metabolomic data were subjected to orthogonal partial least squares discriminant analysis (OPLS-DA) using the ropls package in R language (https://www.r-project.org/, accessed on 27 May 2024). The ShortTime-Series Expression Miner (STEM) software (https://www.cs.cmu.edu/~jernst/stem/, accessed on 27 May 2024) was utilized to analyze the trend of identified and named metabolite abundances in the samples from each group. Clustered profiles with a *p*-value < 0.05 were considered statistically significant. Subsequently, the different metabolites within these profiles were subjected to KEGG pathway enrichment analysis.

### 2.7. Integration of Immune Data or Microbiota and Metabolites

The Pearson correlation coefficients were calculated between the different levels of immune data and metabolomic datasets, as well as between microbiota and metabolomic datasets, using the psych package (version 1.8.4) in R. A correlation heatmap was then generated to visualize these correlations by utilizing the pheatmap package (version 1.0.12) in R. Furthermore, a network analysis was conducted using Cytoscape (version 3.8.2). Linear regression analyses were performed using GraphPad Prism 6.

### 2.8. Statistical Analysis

Data analysis was performed using SPSS 23.0 software. Duncan’s multiple comparisons test in one-way analysis of variance (ANOVA) was used to analyze variables such as villus height, crypt depth, villus/crypt ratio, inflammatory cytokine, and tight junction protein mRNA expression. The results were presented as “Mean ± SEM”. *p* < 0.05 was considered statistically significant, and *p* < 0.01 was considered extremely significant.

## 3. Results

### 3.1. Intestinal Histological Changes during the FIM Process

To quantify the histological damage of the jejunum and ileum during the FIM process, the morphology was assessed using H&E staining. During the fasting period (F3 period and F16 period), extensive damage was observed in the jejunum and ileum (Figure 1B), including a significant decrease in the villus height (*p* < 0.05) (Figure 1C,F) and villus/crypt ratio (*p* < 0.05) (Figure 1E,H), while no significant differences were found in crypt depth (Figure 1D,G). During the refeeding period (R6 period and R32 period), the jejunum and ileum gradually started to recover from injury (Figure 1C,E,F,H). Therefore, fasting led to the injury of the intestinal mucosa.

### 3.2. Change of Intestinal Immune Regulation during the FIM Process

**Figure 2:**
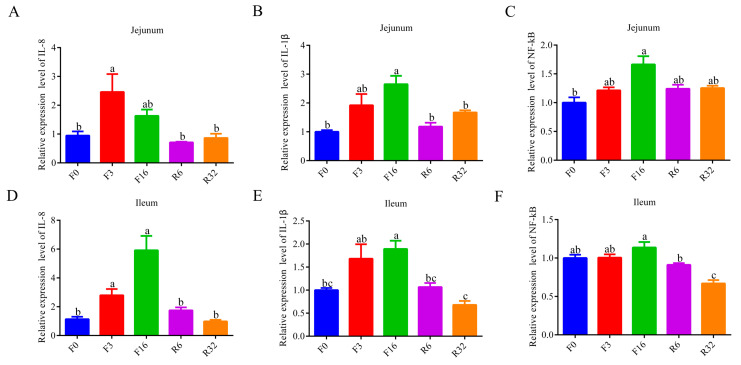
Change in intestinal inflammatory factors during the FIM process. (**A**–**C**) Jejunal mucosa mRNA expression of *IL-8* (**A**), *IL-1β* (B), and *NF-KB1* (**C**). (**D**–**F**) Ileal mucosa mRNA expression of *IL-8* (**D**), *IL-1β* (**E**), and *NF-kB* (**F**). The data were shown as the mean ± SEM. Data with different superscript letters (a, b, c) were significantly different (*p* < 0.05).

Proinflammatory factors, which play important roles in the immune response, were determined in this study. During the fasting period, the expression of *IL-8* and *IL-1β* increased in jejunum and ileum (*p* < 0.05) (Figure 2A,B,D,E); the expression of *NF-kB1* increased in jejunum (*p* < 0.05) (Figure 2C), but there was no significant difference in the ileum (*p* > 0.05) (Figure 2F). During the refeeding period, the expression of *IL-8*, *IL-1β*, and *NF-kB1* returned to pre-fasting levels (Figure 2A–E). The data indicated that fasting triggers intestinal inflammation, leading to intestinal immune responses.

### 3.3. Change in Intestine Barrier during the FIM Process

**Figure 3:**
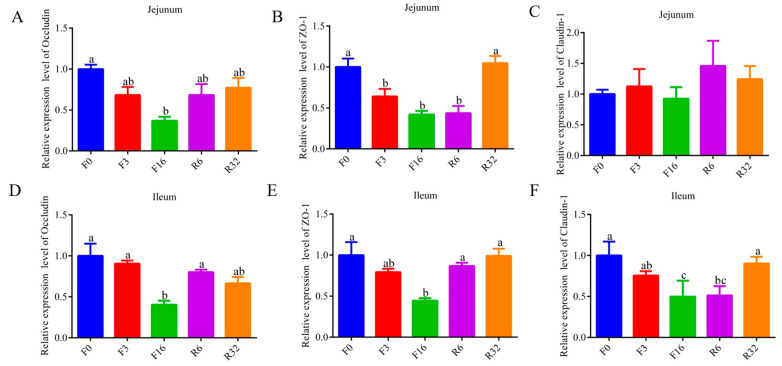
Change in intestine barrier during the FIM process. (**A**–**C**) Jejunal mucosa mRNA expression of *Occludin-1* (**A**), *ZO-1* (**B**), and *Claudin-1* (**C**). (**D**–**F**) Ileal mucosa mRNA expression of *Occludin-1* (**D**), *ZO-1* (**E**), and *Claudin-1* (**F**). Data were shown as the means ± SEM. The data with different superscript letters (a, b, c) were significantly different (*p* < 0.05).

To further verify the change in the intestine barrier during the FIM process, the expression of a tight junction protein was analyzed by qRT-PCR. During the fasting period, the expressions of *Occludin-1* and *ZO-1* were reduced in jejunum and ileum (*p* < 0.05) (Figure 3A,B,D,E); the expression of *Occludin-1* did not reveal a significant difference in jejunum (Figure 3C), but it was reduced in ileum (Figure 3F). During the refeeding period, the expression of *Occludin-1*, *ZO-1,* and Claudin-1 returned to pre-fasting levels (Figure 3A,B,D–F). The data indicated that fasting resulted in elevated intestinal permeability, leading to impairment of intestinal barrier function.

### 3.4. Alteration in the Gut Microbial Composition during the FIM Process

**Figure 4:**
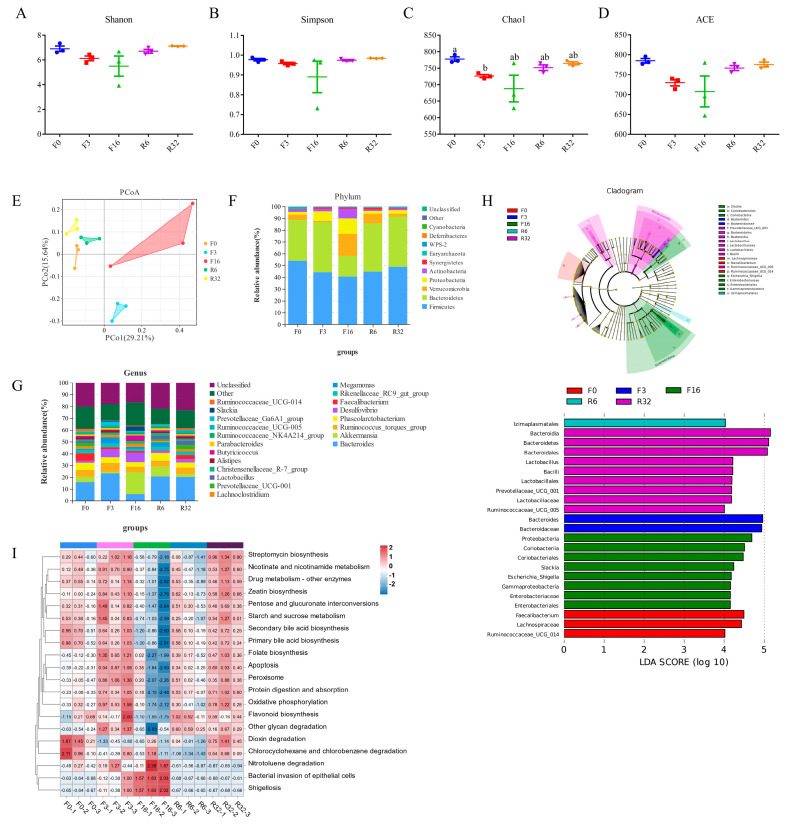
Alteration of the gut microbial composition in laying hens during the FIM process. (**A**−**D**) Alpha-diversity indices. (**E**) PCoA analysis based on unweighted UniFrac metrics of bacterial communities (the difference in PCo1 and PCo2 was 29.21% and 15.64%). (**F**,**G**) The relative abundance of Cecal contents bacterial phylum (**F**) and genus (**G**). (**H**) Analysis of differences in the microbial taxa by LEfSe in different periods (LDA > 4 and *p* < 0.05). (**I**) The heat map of functionals was predicted by PICRUSTt2 analysis.

To comprehensively comprehend gut microbiota dynamics during the FIM process, the gut microbiota was analyzed through 16S rDNA sequencing of cecal contents. The 15 samples collectively obtained 1,967,839 raw sequences. After processing, each sample averaged 111,218 clean tags, including 75,303 being taxon tags. Each sample obtained averaged 688 OTUs, including 27 unique OTUs in the F0 period, 27 in the F3 period, 26 in the F16 period, 23 in the R6 period, and 39 in the R32 period (Figure S1). The dilution curve results indicate that, with the increase in the number of sequencing reads, the curve gradually levels off (Figure S2). Alpha diversity metrics were used to evaluate the microbial diversity. The Shannon, Simpson, Chao1, and ACE indexes were reduced during the fasting period but returned to pre-fasting levels during the refeeding period (Figure 4A–D). Significant separations of gut microbiota from different periods were observed using a principal coordinate analysis (PCoA) (Figure 4E).

At the phylum level, the microbiota structure was similar across different periods, but the composition of each was distinct. The dominant bacterial community in each period was composed of *Firmicutes*, *Bacteroides*, *Verrucomicrobia*, *Proteobacteria,* and *Actinomyces*, which accounted for more than 97% of the total (Figure 4F). During the fasting period, the abundance of *Firmicutes* and *Bacteroidetes* reduced; but the abundance of *Verrucomicrobia* and *Proteobacteria* increased (Figure 4F). During the refeeding period, the abundance of *Firmicutes*, *Bacteroidetes*, *Verrucomicrobia,* and *Proteobacteria* returned to pre-fasting levels (Figure 4F). At the genus level, *Bacteroides*, *Akkermansia*, *Ruminococcus_torques_group*, *Phascolarctobacterium*, *Desulfovibrio*, *Faecalibacterium*, *Lachnoclostridium*, and *Butyricucoccus* were the predominant genera in each period (Figure 4G). During the fasting period, the abundance of *Bacteroides*, *Faeccalibacterium*, *Ruminococcaceae_UCG-014*, *Lactobacillus*, *Ruminococcaceae_UCG-005*, and *Prevotellaceae_UCG-001* reduced (Figure 3A–F); the abundance of *Slackia* and *Escherichia_Shigella* increased (Figure S3G,H) (*p* < 0.05). During the refeeding period, the abundance of *Bacteroides*, *Faeccalibacterium*, *Ruminococcaceae_UCG-014*, *Lactobacillus*, *Ruminococcaceae_UCG-005*, *Prevotellaceae_UCG-001*, *Slackia,* and *Escherichia_Shigella* returned to before fasting levels (Figure S3A–H).

The identification of characteristic bacterial genera was performed using LEFSe to determine differences during the FIM process (Figure 4H). The results showed that *Lachnospiraceae* and *Ruminococcaceae_UCG_014* were enriched during the F0 period; *Bacteroidaceae* were enriched during the F3 period; *Escherichia_Shigella* were enriched during the F16 period; *Izimaplasmatales* were enriched during the R6 period; and *Lactobacillus*, *Prevotellaceae_UCG_005,* and *Prevotellaceae_UCG_001* were enriched during the R32 period (LDA > 4 and *p* < 0.05). The functional profiles of the chicken gut microbiota were examined using PICRUSt2. During the fasting period, the shigellosis and bacterial invasion of epithelial cells pathways were enriched (Figure 4I). During the refeeding period, compared with the F16 period, shigellosis and the bacterial invasion of the epithelial cell pathway were inhibited, while streptomycin biosynthesis, folate biosynthesis, nicotinate and nicotinamide metabolism, apoptosis, drug metabolism, and other enzymes, and the peroxisome pathway were enriched (Figure 4I). The results indicated that pathogenic bacteria increased during the fasting period.

### 3.5. Change in Metabolites during the FIM Process

**Figure 5:**
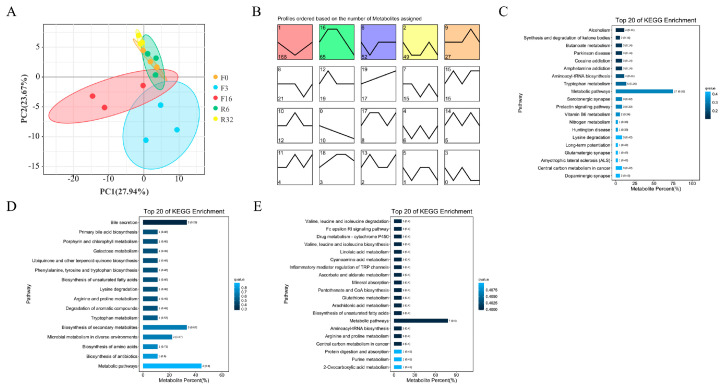
Change in metabolites during the FIM process. (**A**) PCA analysis of cecal metabolites based on Bray distance (the difference in PC1 and PC2 was 38.89% and 12.01%). (**B**) Summary graphs of all trends in differential metabolites. (**C**–**E**) The KEGG enrichment analysis of differential metabolites by trend analysis mode Profile 1 (**C**), Profile 16 (**D**), and Profile 9 (**E**).

Simultaneously, non-targeted metabolomic analysis of the caecum content was carried out during the FIM process. The results of the principal component analysis (PCA) showed that gut metabolites were significantly separated from the F0, R6, R32 period, and the F3, F16 period, suggesting a notable change in gut metabolites during the FIM process (Figure 5A). The subsequent trend analysis was conducted using a total of 499 differential metabolites. Profiles 1, 16, and 9 were selected for the KEGG analysis. Under Profile 1 mode, 168 metabolites exhibited a decreasing trend during the fasting period, which gradually increased during the refeeding period (Figure 5B). These metabolites were enriched in 20 pathways, including Butanoate metabolism, Tryptophan, and the Vitamin B6 metabolism pathway (Figure 5C, Table S2). Under Profile 16 mode, 65 metabolites showed an increase during the fasting period and then a decreasing trend during the refeeding period (Figure 5B). These metabolites were enriched in 16 pathways, including bile secretion, primary bile acid biosynthesis, ubiquinone, and other terpenoid-quinone biosynthesis (Figure 5D, Table S3). Under Profile 9 mode, 27 metabolites showed an upward trend during the fasting period, and the level of metabolites in the offspring of the resumption of feeding decreased gradually (Figure 5B). These metabolites were enriched in 25 pathways, including valine, leucine, and isoleucine degradation, the Fc epsilon RI signaling pathway, the drug metabolism, cytochrome P450, arginine, and proline metabolism (Figure 5E, Table S4). These data suggest that fasting leads to changes in the structure and function of cecum metabolites.

### 3.6. Correlation Analysis of Intestinal Iujury or Genera and Metabolites

**Figure 6:**
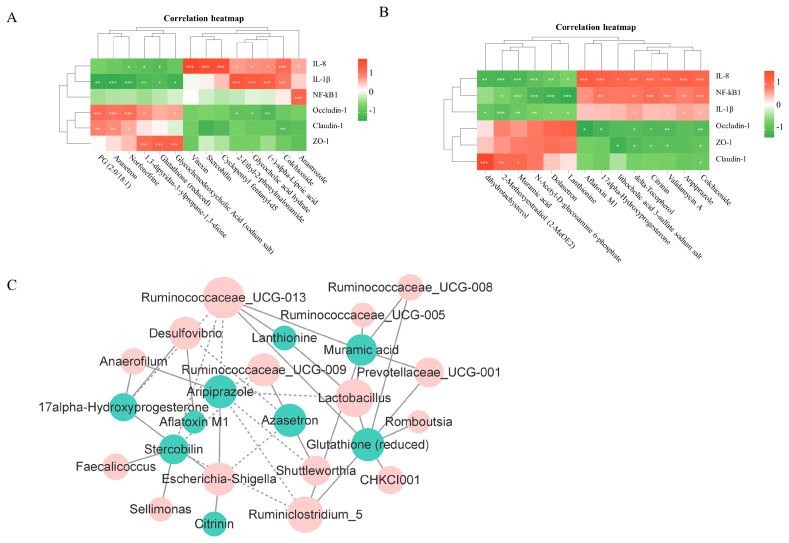
Correlation analysis of intestinal injury or genera and metabolites. (**A**) The correlation heatmap of jejunal injury with cecal metabolites. (**B**) The correlation heatmap of ileal injury with cecal metabolites. Significance levels were defined as follows: * for 0.01 < *p* < 0.05; ** for 0.001 < *p* < 0.01; and *** for *p* ≤ 0.001. (**C**) PPI of correlation analysis of the cecal microbiome and metabolome. Blue represents metabolites; orange represents the microbiome; the full line represents a positive correlation; and the dotted line represents a negative correlation.

To further study whether the changes in intestinal injury were related to intestinal metabolites, the Pearson coefficient was used to evaluate their correlation. The results showed that six metabolites (e.g., Glutathione (reduced)) were negatively correlated with jejunal injury (R < −0.6, *p* < 0.05); seven metabolites (e.g., Stercobilin) were positively correlated with jejunal injury (R > 0.6, *p* < 0.05) (Figure 6A); six metabolites (e.g., Muramic acid and Lanthionine) were negatively correlated with ileal injury (R < −0.6, *p* < 0.05); and eight metabolites (e.g., Aflatoxin M1 and Citrinin) were positively correlated with ileal injury (R > 0.6, *p* < 0.05) (Figure 6B). Based on the cross-check with Pearson’s correlation results, six metabolites were selected to perform linear regression analyses. The results showed that Glutathione (reduced) had a negative linear correlation with jejunal injury (*p* < 0.05); Stercobilin had a positively linear correlation with jejunal injury (*p* < 0.05) (Figure S4A); Muramic acid and Lanthionine had a negative linear correlation with ileal injury (*p* < 0.05); and Aflatoxin M1 and Citrinin had a positively linear correlation with ileal injury (*p* < 0.05) (Figure S4B). Association analysis and linear regression analyses results indicate that Glutathione (reduced), Muramic acid, and Lanthionine are beneficial to intestinal health, while Stercobilin, Aflatoxin M1, and Citrinin are harmful to intestinal health.

To further study whether the changes in intestinal metabolites were related to different genera, the Pearson coefficient was used to evaluate their correlation. The results showed that five genera (e.g., *Escherichia_Shigella*) were positively correlated with metabolites (harmful to intestinal health indicator) (R > 0.7, *p* < 0.05) and ten genera (e.g., *Ruminococcaceae_UCG-0013* and *Lactobacillus*) were positively correlated with metabolites (beneficial to intestinal health indicator) (R > 0.7, *p* < 0.05) (Figure 6C). The linear regression analysis results showed that *Escherichia_Shigella* had a positively linear correlation with Citrinin and Stercobilin (0.1 > *p* > 0.05), *Ruminococcaceae_UCG-0013* had a positively linear correlation with Glutathione (reduced) (*p* < 0.05) and Lanthionine (*p* < 0.05), and *Lactobacillus* had a positively linear correlation with Glutathione (reduced) (*p* < 0.05) and Lanthionine (0.1 > *p* > 0.05) (Figure S5). The data suggest that changes in the microbiota trigger intestinal inflammation through metabolites during the FIM process.

## 4. Discussion

FIM has been shown to extend the laying period of hens, restore egg production rates to their peak, and improve egg quality [3]. Previous studies have primarily focused on enhancing the laying performance of aged laying hens through FIM [6,22]. However, fasting and refeeding treatments have been shown to potentially harm the gut structure and affect the health of laying hens. Therefore, it is of great significance to provide targeted intestinal health programs based on elucidating the intestinal immune mechanisms during the fasting period to alleviate intestinal stress and improve the molting induction technology system, achieving cost savings and increased efficiency. This study found pathological symptoms during the fasting period, including intestinal atrophy, mucosal damage, and an immune response. During the fasting period, the expression of *IL-8*, *IL-1β,* and *NF-kB1* increased in the intestine. The proper functioning of IL-1β, IL-8, and NF-κB1 is essential for the maintenance of intestinal homeostasis and defense mechanisms. Dysregulation of these cytokines can result in the development of inflammatory diseases and the activation of immune responses within the organism [23,24,25,26,27]. During the fasting period, *Occludin1*, *ZO-1*, and *Claudin-1* expressions reduced, leading to increased intestinal permeability and impaired mucosal barrier function [28]. Heightened intestinal permeability can raise the risk of harmful bacteria and substances invading the gut, leading to inflammation. This increase in susceptibility was likely caused by the greater presentation of pathogens and toxins to the immune system as they translocate through defects in the intestinal epithelial barrier [29,30].

At the phylum level, the abundance of *Proteobacteria* increased, while the abundance of *Firmicutes* and *Bacteroidetes* reduced during the fasting period. *Firmicutes* and *Bacteroidetes* are capable of generating short-chain fatty acids (SCFAs) like lactic acid and acetic acid through fermentation [31,32]. Moreover, they regulate bile acid metabolism, collectively contributing to the equilibrium of the intestinal microbiota and playing crucial roles in host health and disease [32,33,34]. In acute colitis models of Min pigs, a reduction in the abundance of *Firmicutes* and *Bacteroidetes* was observed, while *Proteobacteria* and *Spirochaetes* showed an increase compared to control Yorkshire pigs [35]. This suggests a correlation between intestinal inflammation and the reduced abundance of *Firmicutes* and *Bacteroidetes*. In the results, the abundance of *Ruminococcaceae_UCG-014*, *Ruminococcaceae_UCG-005*, and *Lactobacillus* reduced, while the abundance of *Escherichia_Shigella* increased during the fasting period. The genus *Ruminococcus* can ferment dietary fiber and resistant starch to produce short-chain fatty acids (SCFAs), such as butyric acid, propionic acid, and acetic acid [36]. These SCFAs are crucial for maintaining intestinal health as they provide energy to intestinal epithelial cells, promote intestinal barrier function, and have anti-inflammatory effects. *Lactobacillus* plays an important role in maintaining intestinal health by helping to maintain microbial balance, inhibit the growth of harmful bacteria, enhance intestinal mucosal barrier function, and reduce intestinal inflammation [37,38]. *Escherichia_Shigella* is a known pathogen that can infect intestinal epithelial cells and cause the release of pro-inflammatory factors, resulting in inflammation of the intestine [39]. During the fasting period, shigelosis and bacterial invasion of epithelial cells and other pathways were enriched, confirming a significant increase in the abundance of harmful flora during the fasting period. Fasting is likely to have disrupted the intestinal microbiota and epithelial barrier in chickens, which could have resulted in intestinal inflammation.

Intestinal metabolites are essential for preserving the integrity of the intestinal barrier and supporting intestinal homeostasis [40]. During the fasting period, there was inhibition in the Butanoate metabolism, Tryptophan metabolism, and Vitamin B6 metabolism pathways, along with the bile secretion pathway. Butanoate metabolism, Tryptophan metabolism, and Vitamin B6 metabolism pathways are crucial for maintaining intestinal immunity, homeostasis, and epithelial cell structure and function [41,42,43,44,45,46]. Bile acid could promote the digestion, absorption, and transport of cholesterol, triglycerides, and fat-soluble vitamins [47]. However, a high concentration of bile acid has the potential to disrupt the composition of the gut microbiota and also possesses cytotoxic properties that can induce inflammation [48,49]. In the course of the swift recovery phase, there was a notable activation observed in the degradation pathways of valine, leucine, and isoleucine, the Fc epsilon RI signaling pathway, drug metabolism via cytochrome P450, and arginine and proline metabolism. These pathways have the potential to indirectly affect intestinal health by modulating immune function, nutrient utilization, and the composition of the intestinal microbiota [50,51,52,53,54,55]. The research results indicate that fasting can lead to the accumulation of bile acids in the intestines, thereby triggering inflammation. Following refeeding, beneficial metabolites were observed to facilitate the swift recovery of the impaired intestine. Maintaining the balance of intestinal metabolism through external intervention holds significant research value for preserving intestinal health during fasting.

To further investigate the regulatory mechanisms between microbiota, metabolites, and intestinal injury, this study conducted correlation analyses between microbiota and metabolites and between metabolites and immune data. The results indicate that *Escherichia_Shigella* was positively correlated with Citrinin and Sterobilin. Citrinin is toxic to intestinal cells, which may lead to damage to intestinal epithelial cells, disrupt intestinal barrier function, and increase intestinal permeability, thus allowing more toxins and pathogens to pass through the intestinal barrier into the bloodstream [56]. After being absorbed in the intestines, Stercobilin can spread through the bloodstream and may lead to low-level chronic inflammation [57]. Avoiding an elevation in the levels of pathogenic bacteria and their detrimental metabolites is essential for preserving intestinal health while fasting. *Ruminococcaceae_UCG-013* and *Lactobacillus* were positively correlated with Lanthionine and Glutathione(reduced). Lanthionine may affect the intestinal microbiota through its role in cyclic peptide compounds [58]; Glutathione protects intestinal cells through its antioxidative and immune-modulating effects [59]. Lanthionine and Glutathione (reduced) are negatively correlated with intestinal inflammation, while Citrinin and Stercobilin are positively correlated with intestinal inflammation. Therefore, *Ruminococcaceae_UCG-013* and *Lactobacillus* can be used as potential feed additives to improve intestinal health function during fasting and alleviate the stress on the intestines caused by fasting.

## 5. Conclusions

In summary, fasting may lead to an intestinal flora imbalance, reducing some beneficial bacteria while increasing some potentially pathogenic bacteria, thereby impacting intestinal health. The abundance of *Escherichia_Shigella* was increased during the fasting period, while the abundance of *Ruminococcaceae_UCG-013* and *Lactobacillus* was reduced. *Escherichia_Shigella* was positively correlated with Citrinin and Sterobilin, which lead to intestinal inflammation. *Ruminococcaceae UCG-013* and *Lactobacillus* exhibited positive correlations with Lanthionine and Glutathione (reduced), thereby improving intestinal health. *Ruminococcaceae_UCG-013* and *Lactobacillus* can be used as potential additives to enhance intestinal immune function and alleviate the stress on the intestines caused by fasting.

## Data Availability

16S Sequence data that support the findings of this study have been deposited in the National Center for Biotechnology Information with the primary accession code PRJNA1073028. Data are provided within the manuscript or supplementary information files.

## Supplementary Materials

The following supporting information can be downloaded at https://www.mdpi.com/article/10.3390/ani14111640/s1, Figure S1: The VEEN diagram for total OTU number; Figure S2: Rarefaction curve; Figure S3: The abundance of gut microbial composition in genus; Figure S4: Linear regression analyses between metabolite and inflammatory cytokines; Figure S5: Linear regression analyses between metabolite genera and metabolite; Table S1: The primers of qRT-PCR; Table S2: Profile 1 Pathway Enrichment; Table S3: Profile 16 Pathway Enrichment; Table S4: Profile 9 Pathway Enrichment.
