# Supplementary material for: Fasting-Induced Molting Impacts the Intestinal Health by Altering the Gut Microbiota
Source: Animals (Basel). 2024 May 31;14(11):1640. doi: 10.3390/ani14111640 (PMC11171271; doi:10.3390/ani14111640)
Supplement: Supplementary file 1 [file animals-14-01640-s001.zip › animals-3000135-supplementary.pdf]

Supplementary Figures and Table

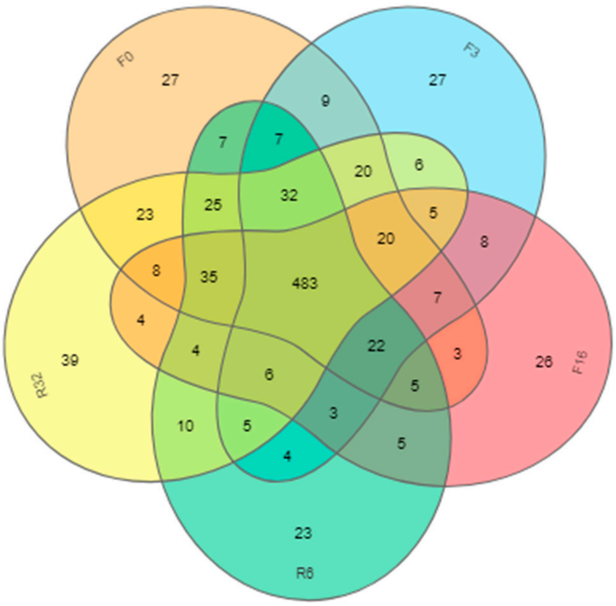

Figure S1. The VEEN diagram for total OTU number.

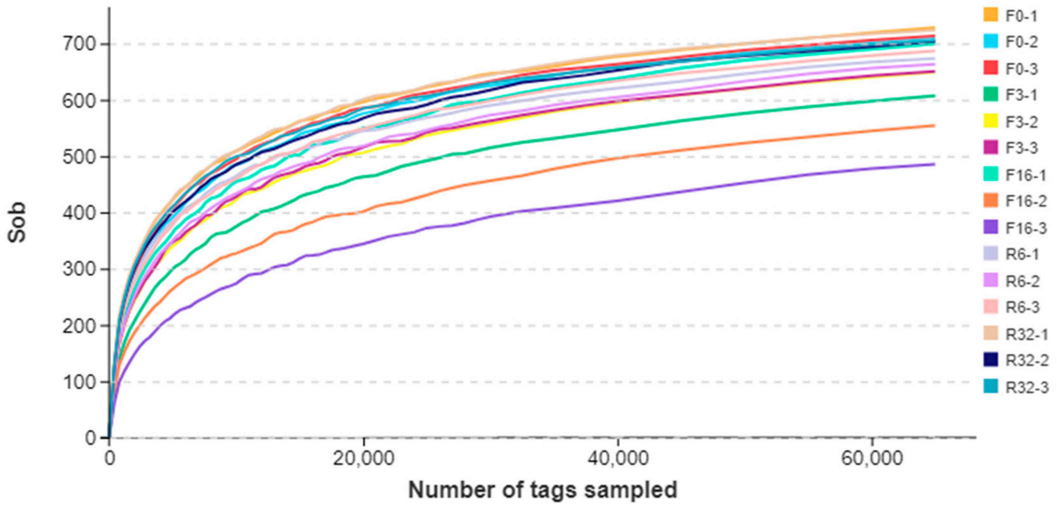

Figure S2. Rarefaction curve.

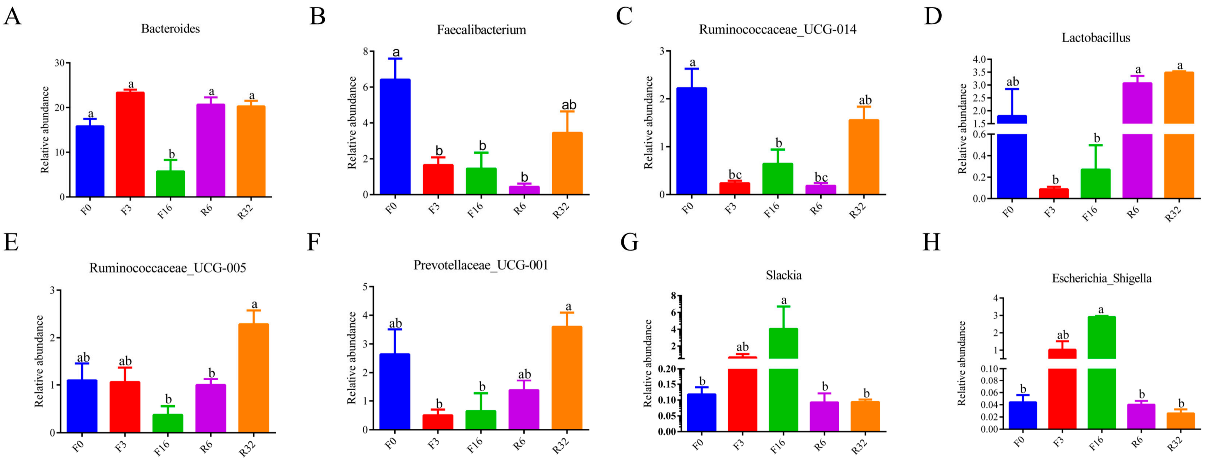

Figure. S3 The abundance of gut microbial composition in genus. A-H In Genus, the abundance of *Bacteriods*, *Faecalibacterium*, *Ruminococcaceae_UCG-014*, *Lactobacillus*, *Ruminococcaceae_UCG-005*, *Prevotellaceae_UCG-001*, *Slackia*, and *Escherichia_shigella*. Data were shown as the mean ± SEM. Data with different superscript letters (a, b, c) were significantly different ($P < 0.05$).

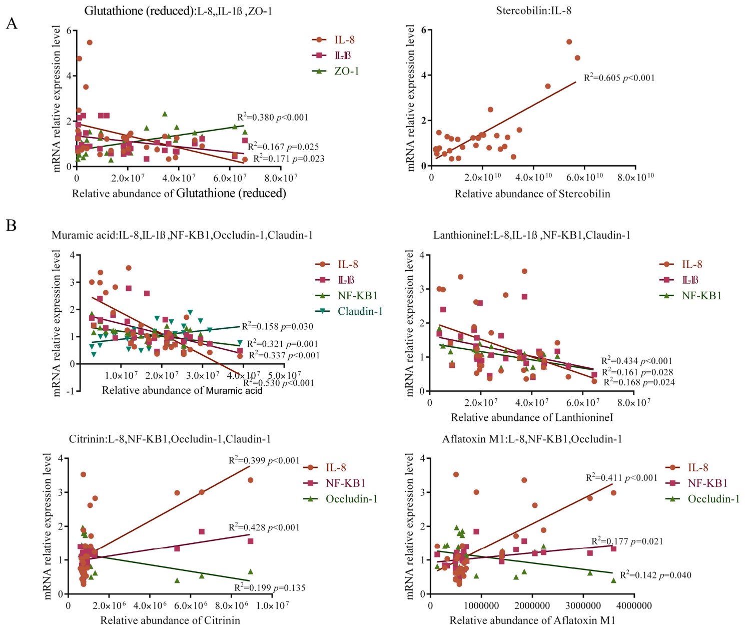

Figure. S4 Linear regression analyses between metabolite and inflammatory cytokines. A Linear regression analyses between metabolite and jejunal inflammatory cytokines. B Linear regression analyses between metabolite and ileal inflammatory cytokines.

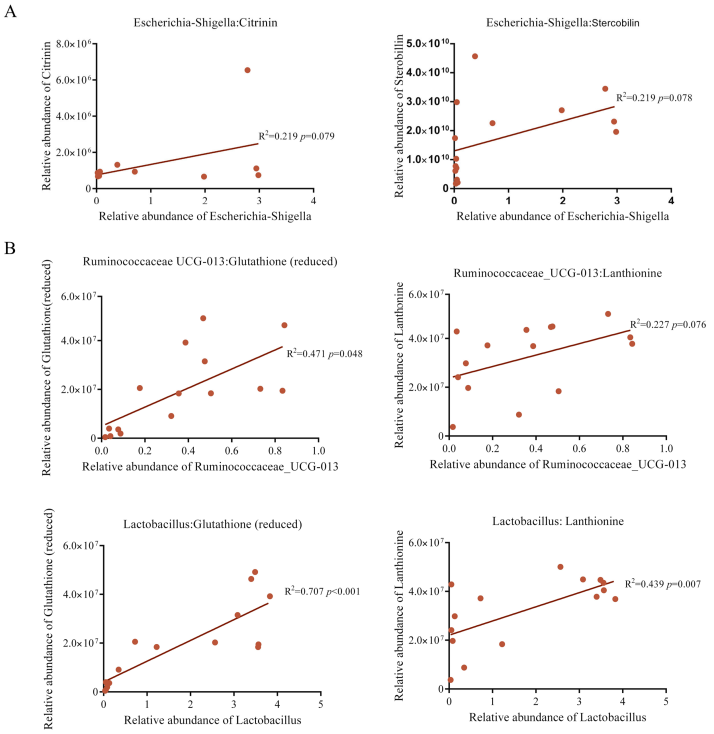

Figure. S5 Linear regression analyses between genera and metabolite.

Table S1. The primers of qRT-PCR

Genes	Primer Sequences（5′→3′）	Tm，℃	Product size, bp
	F: TGCCTGCAGAAGAAGCCTCG	62.79	204
IL-1β	R: GACGGGCTCAAAAACCTCCT	60.25	
	F: CTCTGTCGCAAGGTAGGACG	60.18	240
IL-8	R: GCTGAGCCTTGGCCATAAGT	60.39	
	F: AGAAAAGCTGGGTCTTGGCA	59.81	131
NF-kB	R: CCATCTGTGTCAAAGCAGCG	59.83	
	F: CCGTGGAGTCCAGTGATGAG	59.83	100
Occludin-1	R: TCGAACTCCTGCTTGTAGCG	60.11	
	F: GGTACGAGACCGAAGTGCAA	60.04	155
ZO-1	R: ATATGTGGCTTGCCAACCGT	60.32	
	F: CACCCGTTAACACCAGATTTGA	58.86	156
Claudin-1	R: AGGGGGCATTTTTGGGGTAG	59.96	
	F: GAACATCATCCCAGCGTCCA	60.11	132
GAPDH	R: CGGCAGGTCAGGTCAACAAC	61.51	

Table S2. Profile 1 Pathway Enrichment.

Pathway	Pathway ID	Differentially expressed metabolites	C_id desc
Alcoholism	ko05034	Com_165_neg	L-Glutamate; L-Glutamic acid; L-Glutaminic acid; Glutamate
		Com_295_pos	Adenosine
		Com_5870_pos	3,4-Dihydroxy-L-phenylalanine; L-Dopa; 3-Hydroxy-L-tyrosine; L-beta-(3,4-Dihydroxyphenyl)alanine; Levodopa; Dihydroxy-L-phenylalanine
		Com_4794_pos	3,4-Dihydroxy-L-phenylalanine; L-Dopa; 3-Hydroxy-L-tyrosine; L-beta-(3,4-Dihydroxyphenyl)alanine; Levodopa; Dihydroxy-L-phenylalanine
Synthesis and degradation of ketone bodies	ko00072	Com_1549_neg	Acetoacetate; 3-Oxobutanoic acid; beta-Ketobutyric acid; Acetoacetic acid
		Com_1804_neg	(R)-3-Hydroxybutanoate; (R)-3-Hydroxybutanoic acid; (R)-3-Hydroxybutyric acid; D-beta-Hydroxybutyric acid
Butanoate metabolism	ko00650	Com_165_neg	L-Glutamate; L-Glutamic acid; L-Glutaminic acid; Glutamate
		Com_1549_neg	Acetoacetate; 3-Oxobutanoic acid; beta-Ketobutyric acid; Acetoacetic acid
		Com_1804_neg	(R)-3-Hydroxybutanoate; (R)-3-Hydroxybutanoic acid; (R)-3-Hydroxybutyric acid; D-beta-Hydroxybutyric acid
Parkinson disease	ko05012	Com_295_pos	Adenosine
		Com_5870_pos	3,4-Dihydroxy-L-phenylalanine; L-Dopa; 3-Hydroxy-L-tyrosine; L-beta-(3,4-Dihydroxyphenyl)alanine; Levodopa; Dihydroxy-L-phenylalanine
		Com_4794_pos	3,4-Dihydroxy-L-phenylalanine; L-Dopa; 3-Hydroxy-L-tyrosine; L-beta-(3,4-Dihydroxyphenyl)alanine; Levodopa; Dihydroxy-L-phenylalanine
Cocaine addiction	ko05030	Com_165_neg	L-Glutamate; L-Glutamic acid; L-Glutaminic acid; Glutamate
		Com_5870_pos	3,4-Dihydroxy-L-phenylalanine; L-Dopa; 3-Hydroxy-L-tyrosine; L-beta-(3,4-Dihydroxyphenyl)alanine; Levodopa; Dihydroxy-L-phenylalanine

		Com_4794_pos	3,4-Dihydroxy-L-phenylalanine; L-Dopa; 3-Hydroxy-L-tyrosine; L-beta-(3,4-Dihydroxyphenyl)alanine; Levodopa; Dihydroxy-L-phenylalanine
Amphetamine addiction	ko05031	Com_165_neg	L-Glutamate; L-Glutamic acid; L-Glutaminic acid; Glutamate
		Com_5870_pos	3,4-Dihydroxy-L-phenylalanine; L-Dopa; 3-Hydroxy-L-tyrosine; L-beta-(3,4-Dihydroxyphenyl)alanine; Levodopa; Dihydroxy-L-phenylalanine
		Com_4794_pos	3,4-Dihydroxy-L-phenylalanine; L-Dopa; 3-Hydroxy-L-tyrosine; L-beta-(3,4-Dihydroxyphenyl)alanine; Levodopa; Dihydroxy-L-phenylalanine
Aminoacyl-tRNA biosynthesis	ko00970	Com_165_neg	L-Glutamate; L-Glutamic acid; L-Glutaminic acid; Glutamate
		Com_75_neg	L-Tryptophan; Tryptophan; (S)-alpha-Amino-beta-(3-indolyl)-propionic acid
		Com_1617_neg	L-Histidine; (S)-alpha-Amino-1H-imidazole-4-propionic acid
		Com_2422_neg	O-Phospho-L-serine; L-O-Phosphoserine; 3-Phosphoserine; Dexfosfoserine; 3-Phospho-L-serine
Tryptophan metabolism	ko00380	Com_75_neg	L-Tryptophan; Tryptophan; (S)-alpha-Amino-beta-(3-indolyl)-propionic acid
		Com_2382_pos	Indole-3-acetate; Indole-3-acetic acid; (Indol-3-yl)acetate; Indoleacetate; Indoleacetic acid; (1H-indol-3-yl)acetate; IAA
		Com_3046_neg	Quinolinate; Pyridine-2,3-dicarboxylate; Quinolinic acid; 2,3-Pyridinedicarboxylic acid
		Com_525_pos	5-Hydroxyindoleacetate
		Com_1104_neg	Picolinic acid; 2-Pyridinecarboxylic acid
Metabolic pathways	ko01100	Com_165_neg	L-Glutamate; L-Glutamic acid; L-Glutaminic acid; Glutamate
		Com_75_neg	L-Tryptophan; Tryptophan; (S)-alpha-Amino-beta-(3-indolyl)-propionic acid
		Com_1617_neg	L-Histidine; (S)-alpha-Amino-1H-imidazole-4-propionic acid
		Com_1549_neg	Acetoacetate; 3-Oxobutanoic acid; beta-Ketobutyric acid; Acetoacetic acid
		Com_295_pos	Adenosine
		Com_961_neg	Thymidine; Deoxythymidine
		Com_5162_pos	Pyridoxal

		Com_5870_pos	3,4-Dihydroxy-L-phenylalanine; L-Dopa; 3-Hydroxy-L-tyrosine; L-beta-(3,4-Dihydroxyphenyl)alanine; Levodopa; Dihydroxy-L-phenylalanine
		Com_4794_pos	3,4-Dihydroxy-L-phenylalanine; L-Dopa; 3-Hydroxy-L-tyrosine; L-beta-(3,4-Dihydroxyphenyl)alanine; Levodopa; Dihydroxy-L-phenylalanine
		Com_2038_pos	Ubiquinone; Coenzyme Q; CoQ; Q
		Com_4093_neg	N-Acetylornithine; N2-Acetyl-L-ornithine
		Com_5163_pos	N6-(L-1,3-Dicarboxypropyl)-L-lysine; Saccharopine; L-Saccharopine; N-[(S)-5-Amino-5-carboxypentyl]-L-glutamic acid
		Com_3341_neg	Estrone; 3-Hydroxy-1,3,5(10)-estratrien-17-one
		Com_1388_pos	Allantoate; Allantoic acid
		Com_3972_pos	Pyridoxamine; PM
		Com_3600_pos	4-Hydroxybenzaldehyde; p-Hydroxybenzaldehyde
		Com_6729_pos	Cortisol; Hydrocortisone; 11beta,17alpha,21-Trihydroxy-4-pregnene-3,20-dione; Kendall's compound F; Reichstein's substance M
		Com_2382_pos	Indole-3-acetate; Indole-3-acetic acid; (Indol-3-yl)acetate; Indoleacetate; Indoleacetic acid; (1H-indol-3-yl)acetate; IAA
		Com_2422_neg	O-Phospho-L-serine; L-O-Phosphoserine; 3-Phosphoserine; Dexfosfoserine; 3-Phospho-L-serine
		Com_1804_neg	(R)-3-Hydroxybutanoate; (R)-3-Hydroxybutanoic acid; (R)-3-Hydroxybutyric acid; D-beta-Hydroxybutyric acid
		Com_3046_neg	Quinolinate; Pyridine-2,3-dicarboxylate; Quinolinic acid; 2,3-Pyridinedicarboxylic acid
		Com_1361_neg	Deoxyinosine
		Com_7865_pos	5,6-Dihydroxyindole; DHI
		Com_525_pos	5-Hydroxyindoleacetate
		Com_9020_pos	Thromboxane B2; TXB2
		Com_6206_pos	Fluorene; Diphenylenemethane; 2,2'-Methylenebiphenyl
		Com_1104_neg	Picolinic acid; 2-Pyridinecarboxylic acid
Serotonergic synapse	ko04726	Com_75_neg	L-Tryptophan; Tryptophan; (S)-alpha-Amino-beta-(3-indolyl)-propionic acid
		Com_525_pos	5-Hydroxyindoleacetate

		Com_9020_pos	Thromboxane B2; TXB2
Prolactin signaling pathway	ko04917	Com_5870_pos	3,4-Dihydroxy-L-phenylalanine; L-Dopa; 3-Hydroxy-L-tyrosine; L-beta-(3,4-Dihydroxyphenyl)alanine; Levodopa; Dihydroxy-L-phenylalanine
		Com_4794_pos	3,4-Dihydroxy-L-phenylalanine; L-Dopa; 3-Hydroxy-L-tyrosine; L-beta-(3,4-Dihydroxyphenyl)alanine; Levodopa; Dihydroxy-L-phenylalanine
		Com_3341_neg	Estrone; 3-Hydroxy-1,3,5(10)-estratrien-17-one
Vitamin B6 metabolism	ko00750	Com_5162_pos	Pyridoxal
		Com_3972_pos	Pyridoxamine; PM
Nitrogen metabolism	ko00910	Com_165_neg	L-Glutamate; L-Glutamic acid; L-Glutaminic acid; Glutamate
Huntington disease	ko05016	Com_165_neg	L-Glutamate; L-Glutamic acid; L-Glutaminic acid; Glutamate
Lysine degradation	ko00310	Com_1549_neg	Acetoacetate; 3-Oxobutanoic acid; beta-Ketobutyric acid; Acetoacetic acid
		Com_5163_pos	N6-(L-1,3-Dicarboxypropyl)-L-lysine; Saccharopine; L-Saccharopine; N-[(S)-5-Amino-5-carboxypentyl]-L-glutamic acid
		Com_2943_pos	N6,N6,N6-Trimethyl-L-lysine
Long-term potentiation	ko04720	Com_165_neg	L-Glutamate; L-Glutamic acid; L-Glutaminic acid; Glutamate
Glutamatergic synapse	ko04724	Com_165_neg	L-Glutamate; L-Glutamic acid; L-Glutaminic acid; Glutamate
Amyotrophic lateral sclerosis (ALS)	ko05014	Com_165_neg	L-Glutamate; L-Glutamic acid; L-Glutaminic acid; Glutamate
Central carbon metabolism in cancer	ko05230	Com_165_neg	L-Glutamate; L-Glutamic acid; L-Glutaminic acid; Glutamate
		Com_75_neg	L-Tryptophan; Tryptophan; (S)-alpha-Amino-beta-(3-indolyl)-propionic acid
		Com_1617_neg	L-Histidine; (S)-alpha-Amino-1H-imidazole-4-propionic acid

Dopaminergic synapse	ko04728	Com_5870_pos	3,4-Dihydroxy-L-phenylalanine; L-Dopa; 3-Hydroxy-L-tyrosine; L-beta-(3,4-Dihydroxyphenyl)alanine; Levodopa; Dihydroxy-L-phenylalanine
		Com_4794_pos	3,4-Dihydroxy-L-phenylalanine; L-Dopa; 3-Hydroxy-L-tyrosine; L-beta-(3,4-Dihydroxyphenyl)alanine; Levodopa; Dihydroxy-L-phenylalanine

Table S3. Profile 16 Pathway Enrichment.

Pathway	Pathway ID	Differentially expressed metabolites	C_id desc

Bile secretion	ko04976	Com_153_neg	Cholic acid; Cholate; 3alpha,7alpha,12alpha-Trihydroxy-5beta-cholanate; 3alpha,7alpha,12alpha-Trihydroxy-5beta-cholanic acid
		Com_5_neg	Lithocholic acid; Lithocholate; 3alpha-Hydroxy-5beta-cholanate; 3alpha-Hydroxy-5beta-cholanic acid
		Com_42_neg	Deoxycholic acid; Deoxycholate; 3alpha,12alpha-Dihydroxy-5beta-cholanate; 3alpha,12alpha-Dihydroxy-5beta-cholanic acid
Primary bile acid biosynthesis	ko00120	Com_153_neg	Cholic acid; Cholate; 3alpha,7alpha,12alpha-Trihydroxy-5beta-cholanate; 3alpha,7alpha,12alpha-Trihydroxy-5beta-cholanic acid
Porphyrin and chlorophyll metabolism	ko00860	Com_500_pos	Biliverdin; Biliverdin IX alpha
Galactose metabolism	ko00052	Com_354_pos	N-Acetyl-D-galactosamine; N-Acetyl-D-chondrosamine; 2-Acetamido-2-deoxy-D-galactose
Ubiquinone and other terpenoid-quinone biosynthesis	ko00130	Com_4138_pos	delta-Tocopherol
Phenylalanine, tyrosine and tryptophan biosynthesis	ko00400	Com_2469_pos	Anthranilate; Anthranilic acid; o-Aminobenzoic acid; Vitamin L1; 2-Aminobenzoate
Biosynthesis of unsaturated fatty acids	ko01040	Com_402_neg	Adrenic acid; 7,10,13,16-Docosatetraenoic acid; (7Z,10Z,13Z,16Z)-Docosa-7,10,13,16-tetraenoic acid; 7Z,10Z,13Z,16Z-Docosatetraenoic acid
Lysine degradation	ko00310	Com_256_neg	5-Aminopentanoate; 5-Aminopentanoic acid; 5-Aminovaleric acid
Arginine and proline metabolism	ko00330	Com_256_neg	5-Aminopentanoate; 5-Aminopentanoic acid; 5-Aminovaleric acid
Degradation of aromatic compounds	ko01220	Com_2469_pos	Anthranilate; Anthranilic acid; o-Aminobenzoic acid; Vitamin L1; 2-Aminobenzoate
Tryptophan metabolism	ko00380	Com_2469_pos	Anthranilate; Anthranilic acid; o-Aminobenzoic acid; Vitamin L1; 2-Aminobenzoate
Biosynthesis of secondary metabolites	ko01110	Com_2469_pos	Anthranilate; Anthranilic acid; o-Aminobenzoic acid; Vitamin L1; 2-Aminobenzoate
		Com_500_pos	Biliverdin; Biliverdin IX alpha
		Com_4138_pos	delta-Tocopherol
Microbial metabolism in diverse environments	ko01120	Com_2469_pos	Anthranilate; Anthranilic acid; o-Aminobenzoic acid; Vitamin L1; 2-Aminobenzoate

		Com_256_neg	5-Aminopentanoate; 5-Aminopentanoic acid; 5-Aminovaleric acid
Biosynthesis of amino acids	ko01230	Com_2469_pos	Anthranilate; Anthranilic acid; o-Aminobenzoic acid; Vitamin L1; 2-Aminobenzoate
Biosynthesis of antibiotics	ko01130	Com_2469_pos	Anthranilate; Anthranilic acid; o-Aminobenzoic acid; Vitamin L1; 2-Aminobenzoate
Metabolic pathways	ko01100	Com_2469_pos	Anthranilate; Anthranilic acid; o-Aminobenzoic acid; Vitamin L1; 2-Aminobenzoate
		Com_500_pos	Biliverdin; Biliverdin IX alpha
		Com_153_neg	Cholic acid; Cholate; 3alpha,7alpha,12alpha-Trihydroxy-5beta-cholanate; 3alpha,7alpha,12alpha-Trihydroxy-5beta-cholanic acid
		Com_4138_pos	delta-Tocopherol

Table S4: Profile 9 Pathway Enrichment

Pathway	Pathway ID	Differentially expressed metabolites	C_id desc
Valine, leucine and isoleucine degradation	ko00280	Com_13361_pos	L-Valine; 2-Amino-3-methylbutyric acid
Fc epsilon RI signaling pathway	ko04664	Com_13418_pos	Leukotriene E4; LTE4; (7E,9E,11Z,14Z)-(5S,6R)-6-(Cystein-S-yl)-5-hydroxyeicosa-7,9,11,14-tetraenoate; (7E,9E,11Z,14Z)-(5S,6R)-6-(Cystein-S-yl)-5-hydroxyicosa-7,9,11,14-tetraenoate
Drug metabolism - cytochrome P450	ko00982	Com_10457_pos	2-Propylglutaric acid; 2-PGA
Valine, leucine and isoleucine biosynthesis	ko00290	Com_13361_pos	L-Valine; 2-Amino-3-methylbutyric acid
Linoleic acid metabolism	ko00591	Com_96_neg	Dihomo-gamma-linolenate; Dihomo-gamma-linolenic acid; (8Z,11Z,14Z)-Icosatrienoic acid; (Z,Z,Z)-8,11,14-Eicosatrienoic acid; (Z,Z,Z)-8,11,14-Icosatrienoic acid; (Z,Z,Z)-8,11,14-

			Eicosatrienoate; (Z,Z,Z)-8,11,14-Icosatrienoate; 8,11,14-Eicosatrienoate; 8,11,14-Icosatrienoate; 8Z,11Z,14Z-Eicosatrienoic acid; (8Z,11Z,14Z)-Icosa-8,11,14-trienoic acid
Cyanoamino acid metabolism	ko00460	Com_13361_pos	L-Valine; 2-Amino-3-methylbutyric acid
Inflammatory mediator regulation of TRP channels	ko04750	Com_17381_pos	Cinnamaldehyde
Ascorbate and aldarate metabolism.	ko00053	Com_515_neg	Threonate; L-Threonate; (2R,3S)-2,3,4-Trihydroxybutanoic acid
Mineral absorption	ko04978	Com_13361_pos	L-Valine; 2-Amino-3-methylbutyric acid
Pantothenate and CoA biosynthesis	ko00770	Com_13361_pos	L-Valine; 2-Amino-3-methylbutyric acid
Glutathione metabolism	ko00480	Com_3041_pos	(5-L-Glutamyl)-L-amino acid; L-gamma-Glutamyl-L-amino acid; alpha-(gamma-L-Glutamyl)-L-amino acid
Arachidonic acid metabolism	ko00590	Com_13418_pos	Leukotriene E4; LTE4; (7E,9E,11Z,14Z)-(5S,6R)-6-(Cystein-S-yl)-5-hydroxyeicosa-7,9,11,14-tetraenoate; (7E,9E,11Z,14Z)-(5S,6R)-6-(Cystein-S-yl)-5-hydroxyicosa-7,9,11,14-tetraenoate
Biosynthesis of unsaturated fatty acids	ko01040	Com_96_neg	Dihomo-gamma-linolenate; Dihomo-gamma-linolenic acid; (8Z,11Z,14Z)-Icosatrienoic acid; (Z,Z,Z)-8,11,14-Eicosatrienoic acid; (Z,Z,Z)-8,11,14-Icosatrienoic acid; (Z,Z,Z)-8,11,14-Eicosatrienoate; (Z,Z,Z)-8,11,14-Icosatrienoate; 8,11,14-Eicosatrienoate; 8,11,14-Icosatrienoate; 8Z,11Z,14Z-Eicosatrienoic acid; (8Z,11Z,14Z)-Icosa-8,11,14-trienoic acid
Metabolic pathways	ko01100	Com_231_neg	Adenine; 6-Aminopurine
		Com_13361_pos	L-Valine; 2-Amino-3-methylbutyric acid
		Com_17381_pos	Cinnamaldehyde
		Com_1817_pos	4-Acetamidobutanoate; N4-Acetylaminobutanoate
		Com_96_neg	Dihomo-gamma-linolenate; Dihomo-gamma-linolenic acid; (8Z,11Z,14Z)-Icosatrienoic acid; (Z,Z,Z)-8,11,14-Eicosatrienoic acid; (Z,Z,Z)-8,11,14-Icosatrienoic acid; (Z,Z,Z)-8,11,14-Eicosatrienoate; (Z,Z,Z)-8,11,14-Icosatrienoate; 8,11,14-Eicosatrienoate; 8,11,14-Icosatrienoate; 8Z,11Z,14Z-Eicosatrienoic acid; (8Z,11Z,14Z)-Icosa-8,11,14-trienoic acid

		Com_3041_pos	(5-L-Glutamyl)-L-amino acid; L-gamma-Glutamyl-L-amino acid; alpha-(gamma-L-Glutamyl)-L-amino acid
		Com_13418_pos	Leukotriene E4; LTE4; (7E,9E,11Z,14Z)-(5S,6R)-6-(Cystein-S-yl)-5-hydroxyeicosa-7,9,11,14-tetraenoate; (7E,9E,11Z,14Z)-(5S,6R)-6-(Cystein-S-yl)-5-hydroxyicosa-7,9,11,14-tetraenoate
Aminoacyl-tRNA biosynthesis	ko00970	Com_13361_pos	L-Valine; 2-Amino-3-methylbutyric acid
Arginine and proline metabolism	ko00330	Com_1817_pos	4-Acetamidobutanoate; N4-Acetylaminobutanoate
Central carbon metabolism in cancer	ko05230	Com_13361_pos	L-Valine; 2-Amino-3-methylbutyric acid
Protein digestion and absorption	ko04974	Com_13361_pos	L-Valine; 2-Amino-3-methylbutyric acid
Purine metabolism	ko00230	Com_231_neg	Adenine; 6-Aminopurine
2-Oxocarboxylic acid metabolism	ko01210	Com_13361_pos	L-Valine; 2-Amino-3-methylbutyric acid
